# Early and very early‐onset schizophrenia compared with adult‐onset schizophrenia: French FACE‐SZ database

**DOI:** 10.1002/brb3.1495

**Published:** 2020-01-07

**Authors:** Nathalie Coulon, Ophélia Godin, Ewa Bulzacka, Caroline Dubertret, Jasmina Mallet, Guillaume Fond, Lore Brunel, Méja Andrianarisoa, George Anderson, Isabelle Chereau, Hélène Denizot, Romain Rey, Jean‐Michel Dorey, Christophe Lançon, Catherine Faget, Paul Roux, Christine Passerieux, Julien Dubreucq, Sylvain Leignier, Delphine Capdevielle, Myrtille André, Bruno Aouizerate, David Misdrahi, Fabrice Berna, Pierre Vidailhet, Marion Leboyer, Franck Schürhoff

**Affiliations:** ^1^ Fondation FondaMental Créteil France; ^2^ INSERM U955 Translational Psychiatry laboratory AP-HP, DHU Pe-PSY Centre Expert Schizophrénie Pôle de Psychiatrie et d'Addictologie des Hôpitaux Universitaires Henri Mondor Université Paris Est Créteil Créteil France; ^3^ INSERM U894 AP‐HP Department of Psychiatry Louis Mourier Hospital Paris Diderot University Sorbonne Paris Cité, Faculté de médecine Colombes France; ^4^ EA 3279 : CEReSS -Centre d'Etude et de Recherche sur les Services de Santé et la Qualité de vie Aix-Marseille Univ, Faculté de Médecine Marseille France; ^5^ CRC Scotland & London, Eccleston Square London UK; ^6^ Clermont‐Ferrand University Hospital EA 7280 Auvergne University, BP 69 Clermont‐Ferrand France; ^7^ INSERM U1028 CNRS UMR 5292 Centre de Recherche en Neurosciences de Lyon, Equipe PSYR2 Centre Hospitalier Le Vinatier, Pole Est Claude Bernard Lyon 1 University Bron Cedex France; ^8^ Department of Psychiatry (AP‐HM) Sainte‐Marguerite University Hospital Marseille France; ^9^ Department of Adult Psychiatry Versailles Hospital Le Chesnay France; ^10^ Psychosocial Rehabilitation Reference Center Alpes Isère Hospital Grenoble France; ^11^ INSERM 1061 University Department of Adult Psychiatry La Colombiere Hospital CHU Montpellier University of Montpellier 1 Montpellier France; ^12^ Department of Adult Psychiatry Charles Perrens Hospital University of Bordeaux Bordeaux France; ^13^ INSERM U1114 Strasbourg University Hospital University of Strasbourg, Federation of Translational Psychiatry Strasbourg France

**Keywords:** adult‐onset schizophrenia, duration of untreated psychosis, early‐onset schizophrenia, symptomatology, very early‐onset schizophrenia

## Abstract

**Objective:**

To compare the clinical symptomatology in patients with *Early‐Onset Schizophrenia* (EOS, *N* = 176), especially the subgroup *Very Early Onset Schizophrenia* (VEOS) and *Adult Onset Schizophrenia* (AOS, *N* = 551).

**Method:**

In a large French multicentric sample, 727 stable schizophrenia patients, classified by age at onset of the disorder, were assessed using standardized and extensive clinical and neuropsychological batteries: AOS with onset ≥ 18 years and EOS with onset < 18 years (including 22 VEOS < 13 years).

**Results:**

The importance of better diagnosing EOS group, and in particularly VEOS, appeared in a longer DUP *Duration of Untreated Psychosis* (respectively, 2.6 years ± 4.1 and 8.1 years ± 5.7 vs. 1.0 years ± 2.5), more severe symptomatology (PANSS *Positive And Negative Syndrome Scale* scores), and lower educational level than the AOS group. In addition, the VEOS subgroup had a more frequent childhood history of learning disabilities and lower prevalence of right‐handedness quotient than the AOS.

**Conclusion:**

The study demonstrates the existence of an increased gradient of clinical severity from AOS to VEOS. In order to improve the prognosis of the early forms of schizophrenia and to reduce the DUP, clinicians need to pay attention to the prodromal manifestations of the disease.

## Graphical abstract

To describe schizophrenia from childhood to adulthood is complex and patients with early‐onset schizophrenia (EOS) remain undiagnosed or misclassified, especially the subgroup with very early‐onset schizophrenia (VEOS). In patients with VEOS, the DUP which exceeds 8 years, almost eight times higher than in patients with AOS, objective the problem of early diagnosis and the need to develop diagnostic programs and management cares.

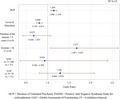

## 1 INTRODUCTION

Classically, schizophrenia often starts in adolescence or in young adults (Chen, Selvendra, Stewart, & Castle, 2018) and is one of the most common and severe forms of mental illness (APA, 2013). It is also very expensive (Laidi et al., 2018; Van Os & Kapur, 2009) for society and is a major public health concern, especially toward an early diagnosis. The *World Health Organization* (WHO) estimated that schizophrenia was the 5th leading worldwide cause of global disease burden (Millier et al., 2014) in 2004 among males, with 2.8% of total *Years Lived With Disability* (YLD), and 6th among females, with 2.6% of YLD. However, it is not easy to describe and define schizophrenia from childhood to adulthood. Since the early 1990s, definitions have tended to harmonize, with *Adult Onset Schizophrenia* (AOS), where the age of onset is greater than or equal 18 years, *Early Onset Schizophrenia* (EOS), defined by an onset strictly before the age of 18 years, and the subgroup *Very Early Onset Schizophrenia* (VEOS), developing strictly before the age of 13 years (Werry, 1992).

In the literature, EOS and VEOS are associated with misdiagnosis and poor outcomes (Asarnow et al., 1994; Gochman, Miller, & Rapoport, 2011; Gordon et al., 1994; Kumra et al., 2000; McKenna et al., 1994; Rapoport & Gogtay, 2011; Rapoport & Inoff‐Germain, 2000; Remschmidt & Theisen, 2012). In particular, according to the neurodevelopmental hypothesis (Gourion et al., 2004; Rapoport & Gogtay, 2011; Sato, Bottlender, Schröter, & Möller, 2004), an earlier age at onset is associated with: more negative symptoms (Rammou et al., 2019); more premorbid deficits (Ballageer, Malla, Manchanda, Takhar, & Haricharan, 2005; Hollis, 2003; Luoma, Hakko, Ollinen, Järvelin, & Lindeman, 2008); higher levels of enuresis (Hollis, 2003); and higher levels of autistic traits (Coulon, 2015). The earlier the schizophrenia begins, the poorer the prognosis and the greater the severity (Coulon, 2015). However, few studies are precise in the definitions with AOS, EOS, and VEOS (see Table 1): In Sato et al. (2004), earlier‐onset schizophrenia was before 40 years and before; Ballageer et al. (2005) compared an onset of psychosis in adolescence (ages 15–18) with young adulthood (ages 19–30); White, Ho, Ward, O'Leary, and Andreasen (2006) studied two groups, one with an onset strictly before the age of 20 and the other with an onset after the age of 20; Schimmelmann, Conus, Cotton, McGorry, and Lambert (2007), Luoma et al. (2008), Joa et al. (2009), and Holmén et al. (2012) studied also two groups with a cutoff close to the age of 18; Biswas, Malhotra, Malhotra, and Gupta (2006, 2007) are the only ones with three groups, but with a cutoff of 14 years for VEOS and small samples sizes (*N* = 35 for EOS and *N* = 20 for AOS). Consequently, available epidemiologic data about VEOS are rare because of differences in definition and VEOS seems to occur very uncommonly in children younger than 13 years. According to several authors (Bleuler, 1972; Lutz, 1972), 4% of schizophrenia begin before age 15 and 0.5%–1% before age 10; Remschmidt, Schulz, Martin, Warnke, and Trott (1994) based on these percentages and estimated that, in the general population, 1 child in 10,000 could develop a schizophrenia, compared to approximatively 1 adult in 100 for this pathology (APA, 2000). In 2011, the NIMH *National Institute of Mental Health* reference team (Gochman et al., 2011) announced a VEOS prevalence of approximately 1 in 40,000. The literature gives moreover some comparisons between AOS and EOS and indicates EOS to have: higher suicidality and more depressive symptoms (Joa et al., 2009), poorer outcomes (Diaz‐Caneja et al., 2015), and longer duration of untreated psychosis (DUP; Joa et al., 2009; Schimmelmann et al., 2007). The DUP is commonly defined as the time interval between onset of positive psychotic symptoms and first adequate treatment (McGlashan, 1999), but DUP according to age at onset in schizophrenia, has rarely been studied (see Table 1), in particular with VEOS. Meta‐analyses and systematic reviews have demonstrated relationship between long DUP and poor outcome in the first year of illness (Marshall et al., 2005; Perkins, Gu, Boteva, & Lieberman, 2005), as well as in longer‐term outcome (Penttila, Jaaskelainen, Hirvonen, Isohanni, & Miettunen, 2014; Perkins et al., 2005). Boonstra et al. (2012) highlighted less severe negative symptoms at short and long‐term follow‐up, especially when the DUP is less than nine months. Consequently, an examination of the DUP, and its reduction (an international goal in World Health Organization, 2011), may have important implications for the clinical management of patients (Fond et al., 2018). Finally, the study of the age at onset could also be used to identify schizophrenia subtypes (Gordon et al., 1994; Werry, 1992) thus distinguishing more homogeneous subgroups of patients, especially in genetic studies (Ahn, An, Shugart, & Rapoport, 2016; Rapoport & Inoff‐Germain, 2000; Schürhoff et al., 2004). Overall, identifying differences in the presentation of VEOS, EOS, and AOS may have important implications for a better description of the disease, thereby improving the possibility of precision medicine for clinically defined homogeneous patient subgroups.

**Table 1:** Comparative studies according to age at onset schizophrenia

Name	Origin	Population: age at onset	Methods	Results
Sato et al. (2004)	Munich, Germany	473 neuroleptic naive SCZ (ICD‐10) Two groups/onset: Group 1 < 40 years or younger, *N* = 418 Group 2 > 41 or older, *N* = 55	German instrument = AMDP; GAF	Group 2: lower score on affective flattening/social withdrawal; higher persecution.
Ballageer et al. (2005)	Manitoba, Québec, Canada	242 FEP Two groups/onset: 15–18 years, *N* = 82 19–30 years, *N* = 119	DUP, SAPS, SANS, ESRS, Calgary; Toxic, PAS, GAF	For EOS, longer DUP (mean 103.57 weeks vs. 46.31), higher negative symptoms
White et al. (2006)	Minnesota, Iowa, USA	188 SCZ (DSM III, III‐R) Two groups/onset: Group 1 < 20 years, *N* = 49 Group 2 ≥ 20 years, *N* = 139	CASH, SAPS, SANS, neuropsychological battery	Group 1: longer DUP (mean 2.4 years versus 1.3 years, greater motor deficits, no difference/measure of negative, disorganized, psychotic symptoms.
Biswas et al. (2006)	Chandigarh, India	55 SCZ (ICD 10) Three groups/onset: <14 years Group 1, *N* = 15 14 ≤ Group 2 < 18 years, *N* = 20 Group 3 ≥ 18 years, *N* = 20	IQ, memory and perceptuor evaluation	Greater deficits in VEOS with decreased gradient of severity to EOS and AOS
Biswas et al. (2007)			IRAOS = Instrument for Retrospective Assessment for Onset of SCZ, PANSS, NSS	NSS more frequent in VEOS (100%), and EOS (90%) than in AOS patients (55%).
Schimmelmann et al. (2007)	Australia, Germany, Switzerland	636 FEP (DSM IV) in EPPIC in Melbourne Two groups/onset: Group 1 < 18 years, *N* = 118 Group 2 ≥ 18 y, *N* = 519	SCID, DUP scale, GAF, PAS	Group 1: worse premorbid functioning, longer DUP (median 26.3 weeks vs. 8.7)
Luoma et al. (2008)	Oulu, Finland	98 SCZ (DSM III‐R) 2 groups/onset: 14–22 years, *N* = 52 23–31 years, *N* = 46	OCCPI = Operational Criteria Checklist for Psychotic Illness	For younger patients: inappropriate affects, positive thought disorder, deterioration from premorbid level of function
Joa et al. (2009)	Stavanger, Norway	232 FEP with 145 SCZ (DSM IV) Two groups: Teens < 18 years, *N* = 43 Adults ≥ 18 years, *N* = 189	SCID, PANSS, GAF, PAS, drug abuse	Teens: longer DUP (mean 77 weeks vs. 33.2), worse premorbid functioning, better PANSS cognitive scores and more depressive symptoms.
Holmén et al. (2012)	Oslo, Norway	110 SCZ (DSM IV) Two groups/onset: 12 < Group 1 < 18 years, *N* = 20 18 ≤ Group 2 < 65 years, *N* = 90	SCID, PANSS, PAS, neuropsychological evaluation	Group 1: higher negative PANSS score and worse premorbid adjustment. No difference in executive function between two groups

Abbreviations: AMDT, Arbeitsgemeinschaft für Methodik und Dokumentation in der Psychiatrie; Calgary Depression Scale; CASH, Comprehensive Assessment of Symptoms and Severity; DSM, Diagnostic and Statistical Mental disorders; DUP, Duration of Untreated Psychosis; EPPIC, Early Psychosis Prevention and Intervention Center; ESRS, Extrapyramidal Symptom Rating Scale; FEP, First Episode Psychosis; GAF, Global Assessment of Functioning Scale; ICD‐10, International Classification of Diseases 10th; NSS, Neurological Soft Signs; PANSS, Positive And Negative Syndrome Scale; PAS, Premorbid Adjustment Scale; SANS, Scale for Assessment of Negative Symptoms; SAPS, Scale for Assessment of Positive Symptoms; SCZ, Schizophrenia.

### 1.1 Aims of the study

The objective of the present study was to compare, for the first time with the consensual definition, the clinical presentation of EOS/VEOS with AOS, in a large multicentric sample of stabilized subjects with schizophrenia. We hypothesized that EOS patients, especially VEOS, would have a more severe clinical symptomatology with poorer premorbid adjustment, longer DUP, and lower cognitive functioning, in comparison with AOS patients.

## 2 METHODS

### 2.1 Subjects

The FACE‐SZ (FondaMental Academic Centers of Expertise for Schizophrenia) cohort is based on a French national network of 10 Schizophrenia Expert Centers (Bordeaux, Clermont‐Ferrand, Colombes, Créteil, Grenoble, Lyon, Marseille, Montpellier, Strasbourg, Versailles), set up by a scientific cooperation foundation in France, the FondaMental Foundation (http://www.fondation-fondamental.org) and created by the French Ministry of Research in order to create a platform that links healthcare and research (Schürhoff et al., 2015).

### 2.2 Inclusion criteria

Consecutive clinically stable patients (defined by no hospitalization and no treatment changes during the 4 weeks before evaluation) with a DSM IV‐TR diagnosis of schizophrenia or schizoaffective disorder were included in the study between 2010 and 2017. The sample was divided into three groups of age at onset (AOS, EOS groups, and VEOS subgroup according to classical cutoff values). Age at onset schizophrenia was defined as the age at which the patient first met DSM IV‐TR criteria for schizophrenia. To limit the recall bias, this age was defined with the help of the patient, his/her family, his/her referring psychiatrist, and with the use of medical case notes. All study participants were referred by their general practitioner or psychiatrist, who subsequently received a detailed evaluation report with suggestions for personalized interventions.

### 2.3 Clinical data

Patients were interviewed by members of the specialized multidisciplinary team of the Expert Center. Diagnostic interviews were carried out by two independent psychiatrists of the same center, according to the *Structured Clinical Interview for Mental Disorders* (SCID 2.0; First, Spitzer, Gibbon, & Williams, 1997). Information about age at onset, age at assessment, age at first treatment, duration of the disease, symptomatology, somatic diseases, comorbidities, and treatments was recorded. The DUP was calculated as in the reference article (see above McGlashan, 1999). Symptoms of schizophrenia were assessed using the *Positive and Negative Syndrome Scale for Schizophrenia* (PANSS; Kay, Fiszbein, & Opfer, 1987). The level of depression in schizophrenia was measured with the *Calgary Depression Rating Scale for Schizophrenia* (CDRS; Addington, Addington, Maticka‐Tyndale, & Joyce, 1992). Therapeutic drug classes (first‐generation antipsychotic (FGA)/second‐generation antipsychotic (SGA); antidepressant; anxiolytic; anticholinergic; antimanic agent; hypnotic) were recorded, and the presence of an extrapyramidal syndrome was evaluated with 10 questions of the French version of *Neurological Soft Signs Scale* (Krebs, Gut‐Fayand, Bourdel, Dischamp, & Olié, 2000). We also used the *Edinburgh Handedness Inventory* (Oldfield, 1971), a measurement scale in order to assess the dominance of a person's right or left handedness in everyday activities. This provides a quotient of laterality (positive for right‐handers, negative for left‐handers). Finally, the *Global Functional Assessment Scale* (GAF; Bodlund et al., 1994; Endicott, Spitzer, Fleiss, & Cohen, 1976), which is a DSM IV numerical scale with a range from 0 to 100, was used to assess the participants psychological, social, and professional functioning.

### 2.4 Neuropsychological measures

We used the *Wechsler Adult Intelligence Scale* (Wechsler, 2008), which provides a measure of general intellectual function in older adolescents and adults. As data from both WAIS‐III and WAIS‐IV were available, we applied a correction algorithm to pool the results. Lichtenberger and Kaufman (2009) defined the standard score difference when both versions are administered to the same subjects. To pool the data, we created a fused WAIS FSIQ variable, taking into account this correction. The seven subtest short form (Bulzacka et al., 2016) was used to estimate the Full Scale IQ (FSIQ), comprised of the following subtests and cognitive areas: Picture Completion (visual exploration and detail perception), Digit‐Symbol Coding (visual‐motor coordination, motor, and mental speed), Similarities (abstract verbal reasoning), Arithmetic (mathematical problem solving), Matrix Reasoning (nonverbal abstract problem solving, inductive spatial reasoning), Digit span (attention, working memory, mental control), Information (degree of general information acquired from culture).

We also used the *National Adult Reading Test* (Mackinnon & Mulligan, 2005; Nelson & O'Connell, 1978) which consists of a list of 40 phonetically irregular words that participants were asked to read aloud. The total raw score ranges from 0 to 40 and was included in regression equations to provide an estimate of premorbid intellectual ability.

Finally, the history of learning disabilities was explored during the neuropsychological evaluation by asking a single question: “have you had learning disabilities” with a dichotomous response (yes/no).

### 2.5 Ethical concerns

The study was carried out in accordance with ethical principles for medical research involving humans (WMA, Declaration of Helsinki). All data were collected anonymously. As this study includes data coming from regular care assessments, a nonopposition form was signed by all participants, all being adults at the time of the assessment. A web‐based application was developed to collate assessment data for clinical monitoring ad research purposes. Access to the system was carefully regulated, and approval was obtained from the Committee in charge of the safety of computerized databases (CNIL DR 2012‐157).

### 2.6 Statistical analysis

Subjects were first divided into two groups and compared: AOS, onset at or over the age of 18 and EOS, onset strictly before the age of 18. Second, specific characteristics were sought in the VEOS subgroup (onset strictly before the age of 13), which was initially included within the EOS group. Three groups, VEOS, EOS, and AOS, were therefore compared. Analyses were conducted using SAS (release 9.4; SAS Statistical Institute). Socio‐demographic, treatment, clinical, and neuropsychological characteristics are presented using measures of means and dispersion (standard deviation) for continuous data and frequency distribution for categorical variables. Comparisons between groups were performed using the chi‐square test or Fisher's exact test for categorical variables. Continuous variables were analyzed with Student's *t* tests for normally distributed data and with the Wilcoxon–Mann–Whitney tests in case of normality violation.

Variables significantly associated in univariate analysis were included in the multivariate logistic regression model to estimate the likelihood that VEOS and EOS were associated with each factor. DUP, level of education, duration of the disease ≥ 5 years, PANSS > 70 “*mildly to moderate ill*” (Leucht et al., 2005; Suzuki et al., 2015), and GAF with three steps (100–71 “*no to slight functional impairment*”; 70–51 “*mild/moderate functional impairment*”; and <51 “*serious functional impairment*” APA, 2000) were studied in the model.

To estimate the relationships between age at onset and DUP, and between DUP and PANSS scores, Pearson's correlation coefficient was used for normally distributed data and Spearman's correlation coefficient for non‐normally distributed data.

## 3 RESULTS

A sample of 727 individuals with stable schizophrenia, enrolled in FACE‐SZ cohort, was included in this study. The sample was composed of 539 (74%) males and 188 (26%) females. The mean age at assessment was 32.2 ± 9.7 years, with a mean age at onset of 21.6 ± 6.5 years. First, individuals with early‐onset schizophrenia (EOS, *N* = 176) were compared to individuals with adult‐onset schizophrenia (AOS, *N* = 551). Table 2 shows clinical and neuropsychological characteristics of the sample. Second, we analyzed data from the subsample with very early‐onset schizophrenia, (VEOS, *N* = 22). Clinical and neuropsychological characteristics of the three groups are detailed in Table 3.

**Table 2:** Clinical and neuropsychological characteristics of the patients according to age at onset schizophrenia (EOS with VEOS/AOS)

Characteristics	VEOS + EOS <18 years (*N* = 176)	AOS ≥ 18 years (*N* = 551)	*p* (*α* = 5%)
Type of disorder (*N* = 727)			
Schizophrenia, *N* (%)	135 (76.7)	414 (75.1)	.631 (*χ* ^2^ 0.921)
Schizoaffective disorder, *N* (%)	37 (21.0)	129 (23.4)	
Schizophreniform disorder, *N* (%)	4 (2.3)	8 (1.5)	
Gender			
Men, *N* (%)/Women, *N* (%)	130 (73.9)/46 (26.1)i	409 (74.2)/142 (25.8)	.921 (*χ* ^2^ 0.009)
(♂/♀)	2.8/1	2.9/1	
Characteristics of age			
Age at assessment (mean years ± *SD*)	26.9 ± 7.9	33.9 ± 9.7	<.0001 (*Z* −8.897)
Age at onset schizophrenia (mean years ± *SD*)	15.1 ± 2.5	23.7 ± 5.9	<.0001 (*Z* −20.029)
Age at first treatment (mean years ± *SD*)	17.5 ± 4.1	23.9 ± 6.9	<.0001 (*Z* −15.169)
Duration of the disease (mean years ± *SD*)	11.8 ± 7.8	10.3 ± 8.4	.0042 (*Z* 2.86)
DUP (mean years ± *SD*)	2.6 ± 4.1	1.0 ± 2.5	<.0001 (*Z* 5.401)
Level of education (mean years ± *SD*)	11.1 ± 2.4	12.6 ± 2.8	<.0001 (*Z* −5.767)
Treatment			
Neuroleptic/antipsychotic, *N* (%)	140 (79.5)	447 (81.1)	.644 (*χ* ^2^ 0.214)
Antidepressant	47 (26.7)	141 (25.6)	.769 (*χ* ^2^ 0.086)
Anxiolytic	45 (25.6)	134 (24.3)	.738 (*χ* ^2^ 0.112)
Anticholinergic	34 (19.3)	80 (14.5)	.127 (*χ* ^2^ 2.324)
Antimanic agent	23 (13.1)	82 (14.9)	.551 (*χ* ^2^ 0.355)
Hypnotic	17 (9.7)	48 (8.7)	.701 (*χ* ^2^ 0.147)
Somatic antecedent			
Allergy, *N* (%)	51 (31.1)	144 (27.4)	.363 (*χ* ^2^ 0.829)
Neurological			
Headache, *N* (%)	21 (12.7)	60 (11.7)	.735 (*χ* ^2^ 0.115)
Epilepsy, *N* (%)	8 (4.9)	21 (4.0)	.617 (*χ* ^2^ 0.250)
Cranial trauma, *N* (%)	16 (10.0)	66 (12.8)	.345 (*χ* ^2^ 0.892)
Endocrinological			
Diabetes, *N* (%)	7 (4.3)	16 (3.1)	.455 (*χ* ^2^ 0.557)
Dysthyroidism, *N* (%)	8 (5.0)	19 ( 3.7)	.463 (*χ* ^2^ 0.538)
Dyslipidemia, *N* (%)	13 (8.2.)	81 (15.8)	.015 (*χ* ^2^ 5.923)
Dermatological			
Acne, *N* (%)	32 (19.6)	70 (13.5)	.06 (*χ* ^2^ 3.682)
Eczema, *N* (%)	16 (9.9)	46 (8.9)	.690 (*χ* ^2^ 0.159)
Hair loss, *N* (%)	4 (2.5.)	40 (7.7)	.017 (*χ* ^2^ 5.650)
PANSS			
Positive score (mean ± *SD*)	15.3 ± 5.4	14.6 ± 5.7	.182 (*F* 1.782)
Negative score (mean ± *SD*)	20.9 ± 8.2	19.8 ± 8.0	.017 (*Z* 2.39)
Psychopathology general score (mean ± *SD*)	37.4 ± 10.3	35.1 ± 10.3	.005 (*Z* 2.84)
Total score (mean ± *SD*)	74.4 ± 18.7	70.2 ± 19.5	.013 (*F* 6.203)
Calgary Scale (mean ± *SD*)	3.9 ± 4.0	4.0 ± 4.4	.854 (*Z* 0.18)
GAF (mean ± *SD*)	46.44 ± 13.189	49.48 ± 12.890	.913 (*F* 0.009)
Neurological Soft Signs			
Extrapyramidal score (mean ± *SD*)	0.264 (0.372)	0.261 (0.360)	.876 (*Z* 0.16)
Neuropsychological evaluation			
Handedness quotient (mean ± *SD*)	58.15 ± 55.7	62.22 ± 53.339	.420 (*F* 6.51)
Intellectual quotient 7 subtests WAIS short form (mean ± *SD*)	82.73 ± 15.659	84.36 ± 15.235	.259 (*F* 1.275)
fNART‐based premorbid IQ (mean ± *SD*)	104.13 ± 8.868	104.16 ± 8.210	.963 (*F* 0.002)
Learning disabilities, *n*(%)	19 (12.8)	42 (9.2)	.197 (*χ* ^2^ 1.662)

Abbreviations: AOS, Adult Onset Schizophrenia; DUP, Duration of Untreated Psychosis; EOS, Early Onset Schizophrenia; fNART, French National Adult Reading Test; GAF, Global Assessment of Functioning; IQ Full, Intelligence Quotient; VEOS, Very Early Onset Schizophrenia; WAIS, Wechsler Adult Intelligence Scale.

**Table 3:** Clinical and neurophychological characteristics of the patients according to age at onset schizophrenia (VEOS, EOS, AOS)

Characteristics	VEOS < 13 years (*N* = 22)	13 years ≤ EOS < 18 years (*N* = 154)	AOS ≥ 18 years (*N* = 551)	*p* global (*α* = 5%)	*p* VEOS vs. EOS	*p* VEOS vs. AOS	*p* EOS vs. AOS
Type of disorder (*N* = 727)							
Schizophrenia, *N* (%)	18 (81.8)	117 (76.0)	414 (75.1)	.757	.878	.726	.600
Schizoaffective disorder, *N* (%)	4 (18.2)	33 (21.4)	129 (23.4)				
Schizophreniform disorder, *N* (%)	0	4 (2.6)	8 (1.5)				
Gender							
Men, *N* (%)/Women, *N* (%)	14 (81.8)/8 (18.2)	116 (75.3)/38 (24.7)	409 (74.2)/142 (25.8)	.501	.294	.324	.787
(♂/♀)	1.8 /1	3.1/1	2.9/1				
Characteristics of age							
Age at assessment (mean years ± *SD*)	24.1 ± 6.2	27.3 ± 8.0	33.9 ± 9.7	<.0001	.06	<.0001	<.0001
Age at onset schizophrenia (mean years ± *SD*)	9.55 ± 2.5	15.9 ± 1.2	23.7 ± 5.9	<.0001	<.0001	<.0001	<.0001
Age at first treatment (mean years ± *SD*)	17.1 ± 6.3	17.5 ± 3.7	23.9 ± 6.9	<.0001	.9	<.0001	<.0001
Duration of the disease (mean years ± *SD*)	14.2 ± 6.4	11.5 ± 8.0	10.3 ± 8.4	.0029	.0355	.0035	.0458
DUP (mean years ± *SD*)	8.1 ± 5.7	1.8 ± 3.1	1.0 ± 2.5	<.0001	<.0001	<.0001	<.0004
Level of education (mean years ± *SD*)	10.5 ± 2.4	11.2 ± 2.4	12.6 ± 2.8	<.0001	.29	.002	<.0001
PANSS							
Positive score (mean ± *SD*)	14.9 ± 4.8	15.4 ± 5.5	14.6 ± 5.7	.388	.937	.975	.355
Negative score (mean ± *SD*)	20.7 ± 7.2	20.9 ± 8.4	19.8 ± 8.0	.334	.996	.864	.325
Psychopathology general score (mean ± *SD*)	39.6 ± 8.8	37.1 ± 10.5	35.1 ± 10.3	.021	.556	.120	.085
Total score (mean ± *SD*)	76.2 ± 15.6	74.2 ± 19.1	70.2 ± 19.5	.041	.896	.343	.068
Neuropsychological evaluation							
Handedness quotient (mean ± *SD*)	36.2 ± 68.249	61.29 ± 53.237	62.22 ± 53.339	.121	.076	.048	.797
Intellectual Quotient, 7 subtests WAIS (mean ± *SD*)	83.25 ± 12.965	82.65 ± 16.082	84.36 ± 15.235	.523	.699	.913	.246
Fnart‐based premorbid IQ (mean ± *SD*)	102.37 ± 9.447	104.38 ± 8.790	104.16 ± 8.210	.619	.291	.298	.873
Learning disabilities, *N* (%)	5 (25.0)	14 (10.9)	42 (9.2)	.065	.080	.020	.066

Abbreviations: AOS, Adult Onset Schizophrenia; DUP, Duration of Untreated Psychosis; EOS, Early Onset Schizophrenia; fNART: French National Adult Reading Test; IQ, Intelligence Quotient; VEOS, Very Early Onset Schizophrenia; WAIS, Wechsler Adult Intelligence Scale.

### 3.1 EOS compared to AOS

On the one hand, there was no statistically significant difference between EOS and AOS across a number of variables, including type of disorder (schizophrenia vs. schizoaffective disorder), gender, treatment (psychotropic class), somatic antecedent (allergy, neurological, endocrinological), level of depression, functioning, neuropsychological soft signs, and neuropsychological evaluation (Table 2).

On the other hand, DUP and PANSS scores were more relevant to observe between EOS and AOS. The mean DUP was 1.6 years longer in EOS, versus AOS, group (2.6 ± 4.1 years vs. 1.0 ± 2.5; *p* < .0001). The EOS, versus AOS, group had higher PANSS scores, especially significantly higher PANSS negative score (20.9 ± 8.2 vs. 19.8 ± 8.0, *p* = .017), psychopathology general score (37.4 ± 10.3 vs. 35.1 ± 10.3; *p* = .005), and total score (74.4 ± 18.7 vs. 70.2 ± 19.5; *p* = .013). About the small difference on negative symptoms between EOS and AOS, but significant, the effect size (Cohen's *d*) for this difference is 0.15, which correspond to small effect. Note also that even the PANSS total score was significantly higher for EOS than AOS, the GAF interval was the same (41–50) with a signification of “*serious symptoms or any serious impairment in social, occupational, or school functioning*” (2), regardless of onset schizophrenia.

Educational level was at last significantly lower in the EOS, versus AOS, group (11.1 ± 2.4 years vs. 12.6 ± 2.8 years; *p* < .0001), and even if there was no significant difference in the neuropsychological evaluation between EOS and AOS groups (see Table 2), it was interesting to note that there was no difference in the premorbid IQ scores between the two groups.

### 3.2 VEOS compared to EOS and AOS

The VEOS subgroup had an almost fourfold longer DUP than the EOS group (8.1 ± 5.7 years vs. 1.8 ± 3.1; *p* < .0001) and an eightfold longer DUP than the AOS group (8.1 ± 5.7 years vs. 1.0 ± 2.5; *p* < .0001). The VEOS subgroup, versus EOS and AOS groups, respectively, also had significantly higher PANSS scores for the psychopathology general score (39.6 ± 8.8 vs. 37.1 ± 10.5 and 35.1 ± 10.3; *p* = .021) and total score (76.2 ± 15.6 vs. 74.2 ± 19.1 and 70.2 ± 19.5; *p* = .041; Table 3). Similarly, educational level was significantly lower in the VEOS subgroup than EOS group and AOS group (10.5 ± 2.4 years vs. 11.2 ± 2.4 years and 12.6 ± 2.8 years; *p* < .0001; Table 3). There were no significant differences in neuropsychological scores among the three groups (Table 3), and it was also interesting to note that there was no difference in the premorbid IQ scores between the three groups, although the VEOS subgroup exhibited (a) more evidence of a history of learning disabilities than the AOS group (25% vs. 9.2%; *p* = .020) and (b) lower right‐handedness quotient than the AOS group (36.2 ± 68.25 vs. 62.22 ± 53.34; *p* = .048).

### 3.3 Multivariate analysis

**Figure 1:**
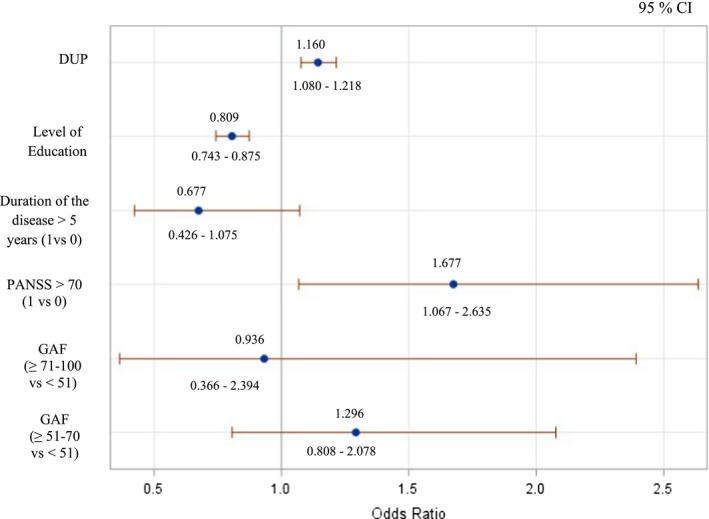
Multivariate analysis, VEOS + EOS <18 years versus AOS ≥ 18 years. The risk of having early onset is significantly associated with higher DUP, lower education level, and higher severity of the disease (PANSS > 70). CI, Confidence Interval; DUP, Duration of Untreated Psychosis; GAF, Global Assessment of Functioning; PANSS, Positive And Negative Syndrome Scale for schizophrenia

After taking into account potential confounders, the risk of having early onset is significantly associated with higher DUP, lower education level, and higher severity of the disease (PANSS > 70). See Figure 1.

### 3.4 Correlation

A negative correlation was found between age at onset and DUP (*ρ* = −226, *p* < .001), while DUP was not correlated to any PANSS scores across all groups (AOS group, EOS group with or without VEOS subgroup, isolated VEOS subgroup, or total population).

## 4 DISCUSSION

The main objective of the present study was to compare the presentation of EOS/VEOS with AOS, making the assumption that EOS patients, especially VEOS, would have a greater severity of symptomatology with poorer premorbid adjustment, a longer DUP, and lower cognitive functioning. The results confirm several of this study's hypotheses and results of univariate analyses are corroborated by multivariate analyses. The EOS group and VEOS subgroup both had more severe symptomatology (especially the general PANSS score), a poorer premorbid adjustment with lower educational level and longer DUP than the AOS group. However, a lower cognitive functioning was not significantly found among the three groups; the VEOS subgroup had only a more frequent history of learning disabilities and lower prevalence of right‐handedness quotient than the AOS.

First, comparisons between EOS and AOS groups showed greater severity in the EOS group, with this EOS group showing longer DUP and higher PANSS scores. Our results are consistent with previous studies carried out in different countries (see Table 1). The longer DUP for the EOS, versus AOS, group: 2.6 years versus. 1.0 year in the current study is similar to previous investigations: a mean 103.57 weeks (2.0 years) versus. 46.31 weeks (0.9 years) in Ballageer et al. (2005); 2.4 years versus. 1.3 years in White et al. (2006); and 77 weeks (1.5 years) versus 33.2 weeks (0.6 years) in Joa et al. (2009). In regard to the PANSS scores, the EOS group had a higher PANSS negative score, compared to the AOS group, which is in accord with the two published studies that have investigated this, namely Ballageer et al. (2005) and Biswas et al. (2007) (Table 1). An increased level of severity on the PANSS psychopathology general score has also previously been found in the EOS, versus AOS group (Biswas et al., 2007). In contrast, no significant difference on PANSS negative score was found according to age at onset in schizophrenia in some previous studies (Biswas et al., 2007; Schimmelmann et al., 2007). In the current study, the PANSS‐positive score showed no significant differences between groups, as in previous studies (Ballageer et al., 2005; Holmén et al., 2012; Joa et al., 2009; White et al., 2006). These data were finally relevant in confrontation with the literature; in particular, in the meaning of the PANSS (Leucht et al., 2005; Suzuki et al., 2015): In 40 articles (*N* = 8,000), cross‐sectional data on the GAF and PANSS (Suzuki et al., 2015) at study baseline or its equivalent were close to our study with a GAF mean score = 49.8 (in our study, 46.22 for EOS group, vs. 49.48 in AOS) and PANSS total score = 76.9 (in our study, 74.4 for EOS group, vs. 70.2 in AOS).

Second, from VEOS to EOS to AOS groups, there was also an increase in severity, with longer DUP (8.1 years vs. 1.8 years and 1.0 years respectively) and more pronounced symptomatology (psychopathology general PANSS score and total PANSS score). Although DUP has been previously investigated in EOS (Diaz‐Caneja et al., 2015; Joa et al., 2009; Stentebjerg‐Olesen, Pagsberg, Fink‐Jensen, Correll, & Jeppesen, 2016), the present study is the first to examine DUP in VEOS patients. This could be partly explained by the difficulty in diagnosing childhood schizophrenia (Gochman et al., 2011). For example, in the first study (McKenna et al., 1994) investigating a cohort of childhood onset schizophrenia (COS), included male and female patients aged 6–18 years, with criteria for schizophrenia and onset at/or before 12 years, the protocol is indicative of the difficulty in the investigation of COS. Following over 450 phone calls, 260 patients were reviewed, with 71 selected and only 19 children (27%) being diagnosed with COS. Moreover in children and teenagers, time is required to check diagnosis reliability as well as to prepare the families for the consequences of the diagnosis. Such factors contribute to the longer DUP, and its associated deleterious consequences (Jeppesen et al., 2008) on relational and functional life in patients and caregivers. Improving early diagnosis in schizophrenia is consequently an important challenge with course and functional implications.

Several countries have developed early intervention programs in order to enhance detection in first‐episode psychosis: USA, RAISE‐ETP (*Recovery After an Initial Schizophrenia Episode–Early Treatment Program*); Australia (EPPIC, *Early Psychosis Prevention and Intervention Centre service*); Canada (PEPP, *Prevention and Early Intervention Program for Psychoses*); Scandinavia (early TIPS *Treatment and Intervention in Psychosis*); Singapore (EPIP, *Early Psychosis Intervention Program*); United Kingdom (STEP‐ED, *Specialized Treatment Early in Psychosis‐Early Detection*). Consequently, there is a growing awareness of this area as a public health problem (Rubio & Correll, 2017).

In the present study, it was hypothesized that an increasing severity would be evident in PANSS negative scores from VEOS to EOS and AOS groups, as seen in Biwas et al. in 2007 (*p* < .001). However, the results did not support this hypothesis. This may be explained by a type II error. The high levels of negative symptoms in EOS and VEOS subgroups could also be due in part, to secondary negative symptoms induced by higher doses of antipsychotic medication, especially when positive symptoms are very present. Conversely, the absence of differences between groups for the positive dimension of the PANSS could be explained by the low temporal stability of this dimension and the Expert Center inclusions were based on clinically stable patients. Previous investigations have showed a relationship between negative symptoms and poor prognosis (Fleischhaker et al., 2005; Vyas, Hadjulis, Vourdas, Byrne, & Frangou, 2007), and more recently the association between negative symptoms and longer DUP (Boonstra et al., 2012; Murru & Carpiniello, 2018; Perkins et al., 2005), so it would be interesting to verify the results by increasing the size of the EOS group/VEOS subgroup. Furthermore, a recent work indicated that aspects of negative symptoms may have a relevant intestinal mucosal immune system impact on their etiology and course, highlighting the importance of a more holistic perspective on the nature of the pathophysiology in schizophrenia (Kanchanatawan et al., 2018).

Thirdly, a lower level of education was found from VEOS to AOS groups (*p* < .0001) and this result could be explained simply by an active onset of the disease earlier, thus disturbing more studies. Nevertheless, there was no significant difference among the three groups regarding neuropsychological functioning (IQ, premorbid IQ). On the one hand, similar inclusion criteria, apart from the age at onset schizophrenia and a relatively similar duration of the disease, could explain these similarities. However, in particular about premorbid IQ, the result might seem counterintuitive because it was more expected a lower premorbid IQ in EOS/VEOS compared with AOS, in relation to higher neurodevelopmental part. With our study, the result might appear to be in favor of a little faster decline in patients with EOS compared with AOS group; hence, the importance of early diagnosis, early management, and more study is needed. On the other hand, in our work, neurocognitive functioning was assessed using a battery of neuropsychological tests; however, we cannot exclude that more accurate tests could lead to the identification of subtle‐specific deficits. In the literature for example, a broad question is if the deficit was present early or was associated with a decline, or a cessation of the development (Kremen et al., 2010). As we observed, Fujino et al. (2017) estimated the cognitive decline in patients with schizophrenia and showed the distribution of approximately 70% of patients had a deterioration. In White et al. (2006), when the developmental course was controlled, there were no significant differences between the adolescent and adult patients' groups in any domains (language, working memory), except in motor function where adolescent performance was worse. In addition, the type II error was also to be considered in our results.

However, despite the small sample, it may be notable that the VEOS subgroup present with: (a) a more extensive history of learning disabilities than AOS (*p* = .020); (b) a lower handedness quotient than AOS (*p* = .048). Even if these results need to be checked on a larger sample, these notions of learning disability and handedness quotient are in agreement with previous data (Alaghband‐rad et al., 1995; Fleischhaker et al., 2005; Remschmidt & Theisen, 2012; Röpcke & Eggers, 2005). In a review of 75 studies (Diaz‐Caneja et al., 2015), the most replicated predictors of clinical, functional, and cognitive outcomes in EOS were premorbid difficulties and symptom severity. Thus, poorer premorbid adjustment from VEOS to EOS and AOS reinforces the importance of early screening and early care. Finally, as previously reported (Biswas et al., 2006; Karp et al., 2001; Kumra et al., 2000), our results support the neurodevelopmental hypothesis in the VEOS group, as indicated by a history of learning disabilities and lower levels of handedness lateralization. About cerebral development in fact, hand preference—or dominance—has been extensively used as an easy‐to‐measure proxy of brain asymmetry, since it has been regarded as a manifestation of cerebral dominance (Sommer, Ramsey, Kahn, Aleman, & Bouma, 2001). The hemispheres of the human brain are anatomically and functionally asymmetric (Gazzaniga, 2005; Oertel‐Knochel & Linden, 2011). The loss of hemispheric lateralization has long been proposed to be a consequence of disrupted neurodevelopment in individuals with psychotic disorder (Oertel‐Knochel & Linden, 2011).

### 4.1 Strengths

The strengths of the study were the use of homogeneous and exhaustive standardized diagnostic protocols and neuropsychological assessments in a large French national multicentric study, as well as the use of hospital records and interviews with patients, their families, and their medical referents, especially important in regard to DUP and illness history. Our study is one of the most important in terms of total sample size (see Table 1), with the largest sample of VEOS published to date with a validated cutoff to define VEOS subgroup. Of note, this is the first study to investigate DUP in VEOS patients. Data on VEOS may contribute more widely to an understanding of the pathophysiology of schizophrenia and therefore to the care provided to patients. Many data about patients with EOS and AOS are in agreement with the literature (PANSS scores, DUP, IQ and premorbid IQ), which guarantees the quality of the study.

### 4.2 Limitations

As the patients have been recruited in different centers, we cannot rule out differences, but meetings allowed us to standardize the quotation between the centers. Particularly in the estimation of the age at onset, we chose clinical evaluation; we did not have an additional and specific tool for this question. To circumvent this point, we used several clinical sources (patient, exchanges with the family, treating psychiatrist, computerized clinical records and files, professional letters) to define the age which the patient first met DSM IV‐TR criteria for schizophrenia. All the psychiatrists had the clinical recommendations of the evaluation for this specific question. Moreover, the recall bias was minimized by a relative homogeneous age at assessment (young adult): Mean age at assessment was younger than 33 years of age, so in general, patients were young enough to still have parents, relatives and a possible computerized file with professional letters. The duration of the disease, between 14.2 years for VEOS and 10.3 years for AOS, also suggests all patients had sufficient insight about the onset of the disease, but not too far away to minimize recall bias. These elements (relative homogeneity of age at assessment and duration of the disease) could also reinforce the estimation of premorbid intellectual ability.

We have a large nonselected national sample of 727 individuals, but with three groups of different size, particularly VEOS subgroup (which also emphasizes the importance of improving the diagnosis of young patients). Therefore, in order to limit statistical errors related to the small VEOS subgroup, we first analyzed the main groups, AOS (*N* = 551) and EOS (*N* = 176) and we secondly conducted an in‐depth examination of the significant results. However, we had to specify this limitation several times in the interpretation of our results (PANSS results, neuropsychological evaluation).

Our data were analyzed in a cross‐sectional design, with a stabilized psychotic phenomenology and require now investigation within a longitudinal design. A stable condition is also important to note for the interpretation of the clinic and neuropsychology and could also mitigate some differences. In all cases, the present results may indicate VEOS to be underdiagnosed and undertreated in child populations, which requires investigation in future studies, as this help to reduce DUP and improve illness prognosis.

In conclusion, the EOS group, especially the VEOS subgroup, exhibited longer DUP, more severe symptomatology and a lower educational level than the AOS group. The study demonstrated the existence of an increased gradient of clinical severity from AOS to VEOS. In order to improve the prognosis of the early forms of schizophrenia and to reduce the DUP, clinicians need to pay attention to the prodromal manifestations of the disease.

Improving early diagnosis and organizing targeted programs may be crucial to the management and outcome of EOS. An innovative and multidisciplinary public health approach may prove of utility, involving care and educational professionals, as well as the patient's family.

## CONFLICT OF INTEREST

The authors have declared that there are no conflicts of interest in relation to the subject of this study.

## AUTHORS' CONTRIBUTION

NC had the idea of the article subject and organized the collection of the data in the Foundation Fondamental with ML and FS. EB gave her expertise for the neuropsychological data. OG made statistical analyses in agreement with NC and FS. NC wrote the draft and the main authors OG, EB, ML and FS fixed it. NC and FS drew up a synthesis and all authors have been significantly involved in the research and/or article preparation. All authors have approved the final article.

## Data Availability

The data that support the findings of this study are the property of the hospital and the Foundation FondaMental. Any request goes through the corresponding author and the Foundation FondaMental. The data are not publicly available due to privacy or ethical restrictions.
